# *Bifidobacterium adolescentis PRL2019* in Pediatric Irritable Bowel Syndrome: A Multicentric, Randomized, Double-Blind, Placebo-Controlled Trial

**DOI:** 10.3390/microorganisms13030627

**Published:** 2025-03-10

**Authors:** Valentina Giorgio, Giovanna Quatrale, Maurizio Mennini, Marisa Piccirillo, Silvia Furio, Giuseppe Stella, Alessandro Ferretti, Pasquale Parisi, Melania Evangelisti, Enrico Felici, Paolo Quitadamo, Giovanni Di Nardo

**Affiliations:** 1 Department of Woman and Child Health and Public Health, Fondazione Policlinico Universitario “A. Gemelli” IRCCS, Catholic University of Sacred Heart, 00168 Rome, Italy; 2 Department of Neurosciences, Mental Health and Sensory Organs (NESMOS), Sapienza University of Rome, 00189 Rome, Italy; 3 Pediatric Unit, Sant’Andrea University Hospital, 00189 Rome, Italy; 4 Pediatric Unit, Children’s Hospital, Azienda Ospedaliera SS Antonio e Biagio e Cesare Arrigo, 15121 Alessandria, Italy; 5 Pediatric Gastroenterology and Hepatology Unit, Santobono-Pausilipon Children’s Hospital, 80122 Naples, Italy

**Keywords:** children, irritable bowel syndrome, gut microbiota, microbiota–gut–brain axis, bifidobacterium adolescentis PRL2019

## Abstract

The gut microbiota plays a pivotal role in gastrointestinal inflammation and immune response since changes in microbiota may result in abnormal neurotransmitter expression, inducing changes in gastrointestinal sensory–motor function and leading to symptom onset in irritable bowel syndrome (*IBS*) patients. The *Bifidobacterium adolescentis* species has a documented immunomodulatory effect through its ability to produce γ-aminobutyric acid (*GABA*), the primary inhibitory neurotransmitter in the mammalian central nervous system, which is reduced in *IBS* patients. This is a multicentric, randomized, double-blind, placebo-controlled, parallel-arm trial aimed at evaluating the effectiveness of *Bifidobacterium adolescentis PRL2019* in children with *IBS. IBS* children diagnosed according to Rome IV criteria were enrolled and randomized into two groups to receive one stick containing 20 × 10^9^ colony-forming unit of *Bifidobacterium adolescentis PRL2019* (Gabapral, Pontenure, Italy) or an equivalent placebo once a day, in a 1:1 ratio, for 12 weeks. Clinical evaluation of symptoms was performed every four weeks using validated scores. Bowel habit characteristics were assessed using the Bristol Stool Chart (*BSC*). Seventy-two subjects (mean age 12.2 ± 1.8 years, 30 males) were enrolled and randomized into two groups, each of thirty-six patients. No significant differences were observed between the two groups regarding demographic characteristics, distribution of *IBS* subtypes, or baseline measures of *IBS* severity and *BSC*. The proportion of patients achieving complete remission was significantly higher in the *BA* Group (19/36; 52.8%) than in the Placebo Group (7/36; 19.4%, *p* = 0.003, odds ratio [OR] 0.216, 95% confidence interval [CI] 0.075–0.619). Both groups obtained a reduction in Total *IBS* Symptom Severity Scale (*IBS SSS*), Pain Intensity Score (*PIS*), Pain Frequency Score (*PFS*), and Life Interference Score (*LIS*) from T0 to T12. However, upon intergroup comparison, only in the *BA* group did the *IBS-SSS* (*p* = 0.001), *PIS* (*p* = 0.001), *LIS* (*p* = 0.015), and *PFS* (*p* = 0.005) significantly improve between T0 and T12. *BSC* showed a greater representation of normal stools (type 3–4) at the end of treatment in the *BA* group compared with baseline (25% vs. 58.3%, *p* = 0.004), especially in patients who presented an *IBS*–constipation subtype at T0 (44.5% vs. 19.4%, *p* = 0.02). In our study, *Bifidobacterium adolescentis PRL2019* reduces the severity and frequency of symptoms in children with *IBS*, positively affecting bowel habits in children with the *IBS*–constipation subtype.

## 1. Introduction

Irritable bowel syndrome (*IBS*) is a functional gastrointestinal (*GI*) disorder (*FGID*) characterized by recurrent episodes of defecation-related abdominal pain associated with abnormal bowel habits [1]. The global prevalence of *IBS* in children varies between 1.2% and 22.6% in countries [2,3,4,5]. Despite its benign nature, *IBS* has a significant deleterious impact on children’s quality of life and might result in considerable psychological and emotional burdens for both children and their families [6]. In the most severe cases, *IBS* is associated with a substantial cost to the healthcare systems due to the significant utilization of healthcare resources [7].

Although the underlying pathophysiologic mechanisms remain incompletely understood, current evidence considers *IBS* as a disorder of the brain–gut axis resulting from interactions among environment, host, and genetic factors. In genetically predisposed individuals, various triggers, such as diet, microbiota, and bile acids, may contribute to the loss of intestinal barrier function, allowing antigens to pass through the mucosal layer [8]. This may elicit mucosal immune responses, primarily through mast cell recruitment and activation, which induce changes in *GI* sensory–motor function, leading to symptom generation in *IBS* patients [9].

Several studies have reported significant alterations in the gut microbiota that may promote *IBS* development [10,11,12,13]. A recent meta-analysis of the molecular signature of intestinal microbiota showed a significantly lower abundance of *Lactobacillus*, *Bifidobacterium*, and *Faecalibacterium prausnitzi*, but not the *Bacteroides-Prevotella* group, *Escherichia coli*, or other species in *IBS* patients [12]. Additional data showed that patients with an *IBS* mixed subtype (*IBS-M*) or an *IBS* diarrhea subtype (*IBS-D*) had a reduction in butyrate-producing bacteria, known to improve intestinal barrier function [13].

*Lactobacillus rhamnosus GG* (*LGG*) has received significant attention among various probiotics studied in children. A meta-analysis of three RCTs on the efficacy of *LGG* for treating abdominal pain-related functional gastrointestinal disorders in childhood found that *LGG* treatment significantly reduced the intensity and frequency of pain in the *IBS* subgroup [14]. One RCT evaluated the effect of *Lactobacillus reuteri DSM 17938* in children with *IBS*. Children in the intervention group had significantly more days free of pain and less severe abdominal pain starting from the second month of treatment [15]. A multicenter international double-masked, placebo-controlled, cross-over RCT examining the effect of the probiotic mixture (*Bifidobacterium breve*, *B. longum*, *B. infantis*, *Lactobacillus acidophilus*, *L. plantarum*, *L. casei*, *L. bulgaris*, *and Streptococcus thermophilus*) was conducted in children and adolescents with *IBS*. The results showed that the probiotic mixture significantly improved the frequency and intensity of abdominal pain over placebo. Similarly, significant benefits were observed for bloating and gas, subjective relief of symptoms, and caregivers’ satisfaction [16].

The efficacy of a mixture of *Bifidobacterium infantis M-631*, *breve M-16V1*, and *longum BB5361* has been assessed in children with *IBS*. The study demonstrated a significant decrease in abdominal pain prevalence and severity and improved quality of life in *IBS* patients treated with probiotics [17].

Based on animal model studies, some *Bifidobacterium* species, mainly *Bifidobacterium adolescentis*, have a documented immunomodulatory effect [18,19] and can modulate visceral hypersensitivity or improve the integrity of the intestinal epithelium barrier through its well-known ability to produce γ-aminobutyric acid (*GABA*) [20,21,22,23]. *GABA* is the main mediator of inhibitory transmission in the mammalian central nervous system, playing a pivotal role in regulating some enteric nervous system (*ENS*) functions, such as intestinal motility and pain perception [24]. Recent studies have shown the alteration of the GABAergic signal system in *IBS-D* patients as compared with controls, resulting in a reduced level of *GABA* in *IBS* patients [25].

Thus, we designed a multicentric randomized, double-blind, placebo-controlled, parallel-arm study evaluating the efficacy of *Bifidobacterium adolescentis PRL2019* on abdominal pain symptoms in pediatric patients with *IBS*.

## 2. Methods

### 2.1. Study Design

This is a multicentric, randomized, double-blind, placebo-controlled, parallel-arm trial aimed at evaluating the efficacy of *Bifidobacterium adolescentis PRL2019* in pediatric patients (>four years) with *IBS* of any subtype within the Rome IV criteria [1].

**Figure 1:**
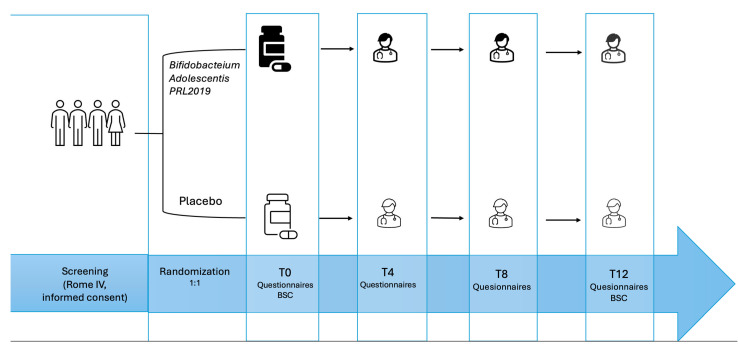
Study design.

All parents/guardians and children (where appropriate) fulfilled an informed consent to enter the study protocol. The study included a 2-week screening period and a 12-week placebo-controlled treatment period (Figure 1).

After the screening phase, eligible patients were randomly assigned to either one oral stick containing 20 × 10^9^ colony-forming units of *Bifidobacterium adolescentis PRL2019* (Gabapral, Pontenure, Italy) or an equivalent placebo, once a day, in a 1:1 ratio, for 12 weeks. Study visits were conducted every four weeks during the treatment period. All the subjects were blindly allocated using scratch cards to one of the two treatment groups according to a computer-generated randomization list provided by our statistician. An independent statistician used a validated program to generate a randomization list with blocks, block size = 4, pre-allocated to centers. Patients and study investigators were blinded to the randomization codes. The codes were kept confidential until the end of the study when the randomization code was broken after the database lock.

Compliance was monitored through monthly phone calls. Patients and patients’ families were also asked to take *Bifidobacterium adolescentis PRL2019* sticks/placebo sticks with them during the visits at 4, 8, and 12 weeks after starting the study. Therefore, treatment compliance was evaluated during every visit by reviewing medication records in a patient’s diary reporting stick count and adverse events, and direct interviews with patients. Compliance was estimated as a percentage of sticks taken during the treatment and greater than 90% in both groups (mean values of 93% vs. 94%).

All subjects underwent a formal clinical assessment and were further characterized using validated questionnaires [26]. Daily bowel movement frequencies were documented, and the Bristol Stool Chart was utilized to evaluate characteristics of bowel habits [27].

All enrolled patients were given traditional dietary advice before starting the study period and at 8 weeks after starting treatment/placebo by a physician. Recommendations included healthy eating and lifestyle management that involved regular meals, adjustment of fiber intake, adequate fluid intake, decreasing fat intake, and assessing components of spicy meals. Eating smaller and more frequent meals was suggested [28]. Indications of how to prepare meals in terms of carbohydrate, fat, and protein distribution were also given, as reported in the Reference Intake Levels of Nutrients and Energy (*LARN*) for the Italian population—4th edition [29].

The protocol has been approved by an independent Ethics Committee of Sant’Andrea University Hospital in Rome (Prot. n°Ped.22.07 del CE 15 December 2022) and conducted according to the Declaration of Helsinki and the principles of good clinical practice. The trial was registered in a public registry (ClinicalTrials.gov ID: NCT05737277).

The primary outcome was the percentage of patients who achieved complete remission, defined as a Total IBS-Symptom Severity Scale (*IBS-SSS*) score of less than 75 points after 12 weeks of therapy [26]. *IBS-SSS* contains the usual demographic information and instructions for the patient on how to use the questionnaire. It includes questions and visual analog scales to assess abdominal symptom intensity and frequency. The last section assesses an overall view of quality of life [26]. Thus, three *IBS-SSS* subscales can be identified: Pain Intensity Score (*PIS*), Life Interference Score (*LIS*), and Pain Frequency Score (*PFS*). Secondary outcome parameters including frequency and severity of abdominal pain symptoms were assessed using *PIS*, *LIS*, and *PFS*, while changes in bowel habits were evaluated using the Bristol Stool Chart (*BSC*).

### 2.2. Study Patients

Eligible patients meeting the Rome IV criteria [1] for *IBS* were recruited from the Outpatients Pediatric Gastrointestinal Unit of three Italian referral centers (Sant’Andrea University Hospital in Rome, Fondazione Policlinico Universitario A. Gemelli IRCCS in Rome, and S. Antonio e Biagio e Cesare Arrigo Hospital in Alessandria).

Inclusion criteria included a positive diagnosis of all *IBS* subtypes (*IBS* with constipation [*IBS-C*], diarrhea [*IBS-D*], mixed bowel habits [*IBS-M*]), an age range of 4–17 years, a negative fecal calprotectin, and negative antitransglutaminase antibodies.

Exclusion criteria included the current use of non-steroidal anti-inflammatory drugs, corticosteroids, mast cell stabilizers, topical or systemic antibiotics in the last month, and the continuous use of stimulant laxatives. Also, the use of any probiotic, prebiotic, or postbiotic in the previous 2 months before enrolment was considered exclusion criteria, as well as any history of major abdominal surgery, Inflammatory Bowel Disease, infectious diarrhea in the last three months, any allergic disease, and any other organic and psychiatric disorders.

## 3. Statistical Analysis

The sample size was calculated based on a 30% difference between the case and placebo groups in achieving complete remission, defined as an *IBS-SSS* score of less than 75 points after 12 weeks of therapy. To achieve a power of 80% with a significance level of 0.05, an average of 30 participants per group was required. To account for a potential 20% dropout rate, it was determined that 36 patients per treatment arm would be sufficient to detect this difference.

The normal distribution of data was assessed using the Kolmogorov-Smirnov test. Accordingly, the values were expressed as a number and percentage (%) for categorical variables, mean ± standard deviation (SD) for normally distributed continuous variables, or median and interquartile range (25−75 percentile) for non-normally distributed continuous variables.

We analyzed effectiveness outcomes in the intention to treat population (ITT), defined as all participants randomly allocated, regardless of adherence. The difference between continuous variables was assessed either by a two-tailed Student *t*-test for values with normal distribution or the Mann–Whitney test for non-normally distributed variables. Based on distribution, the Student *t*-test for paired samples or the Wilcoxon test was used for paired samples. A chi-squared test was used to compare categorical variables. An SPSS software (Version 26, SPSS Inc., Chicago, IL, USA) was used for the statistical analysis. “*p*” values of <0.05 were considered statistically significant.

## 4. Results

**Figure 2:**
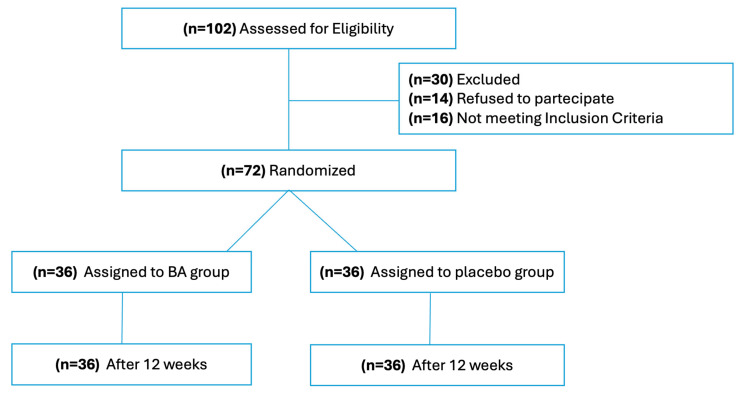
Consort diagram of the study.

We prospectively screened 102 children from January 2023 to January 2024. Of these, 16 were excluded for not meeting the inclusion criteria, and 14 declined to participate in the study (Figure 2).

Seventy-two subjects (mean age 12.2 ± 1.8 years, 30 males) were enrolled and randomized into two groups, each of thirty-six patients. No significant differences were observed between the two groups regarding demographic characteristics, distribution of *IBS* subtypes, or baseline measures of *IBS* severity and *BSC* (Table 1).

**Figure 3:**
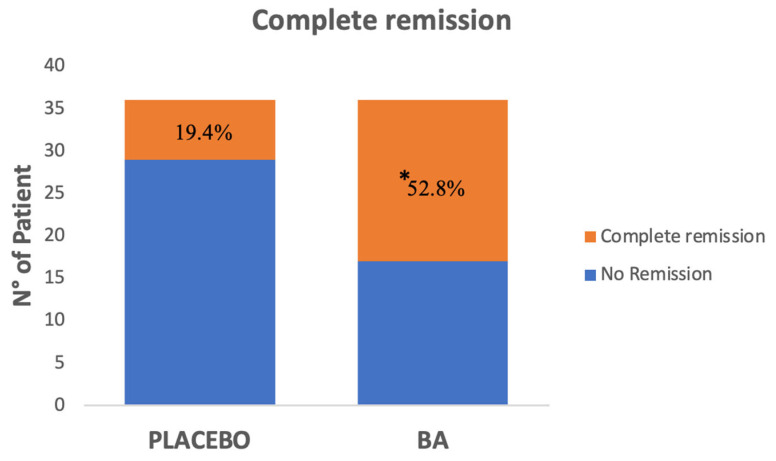
Complete remission in *Bifidobacterium adolescent’s PRL2019* (*BA*) and placebo group * significant difference vs. T0.

In the ITT analysis, the proportion of patients achieving the primary end-point (*IBS-SSS* score <75 points after 12 weeks of therapy) was significantly higher in the *BA* Group (19/36; 52.8%) than in the Placebo Group (7/36; 19.4%, *p* = 0.003, odds ratio [OR] 0.216, 95% confidence interval [CI] 0.075–0.619, Figure 3).

*IBS-SSS*, Pain Intensity Score (*PIS*), Life Interference Score (*LIS*), and Pain Frequency Score (*PFS*) were analyzed in both groups at baseline (T0) and 4, 8, and 12 weeks after treatment/placebo.

Both groups obtained a reduction in Total *IBS-SSS*, Pain Intensity Score, Pain Frequency Score, and Life Interference Score over time. All scores significantly reduced between T0 and T12 (Table 2).

However, upon intergroup comparison, only the *BA* group exhibited a significant reduction in Total *IBS-SSS* at T8 (*p* = 0.016) and T12 (*p* = 0.001), Pain Intensity Scores at T8 (*p* = 0.004) and T12 (*p* = 0.001), and Pain Frequency Scores at T8 (*p* = 0.001) and T12 (*p* = 0.005), as well as LIS at T12 (*p* = 0.015), when compared with the outcomes of Placebo Group (Table 2). Upon intergroup analysis, no significant differences were obtained between the placebo and *BA* group at T4.

**Figure 4:**
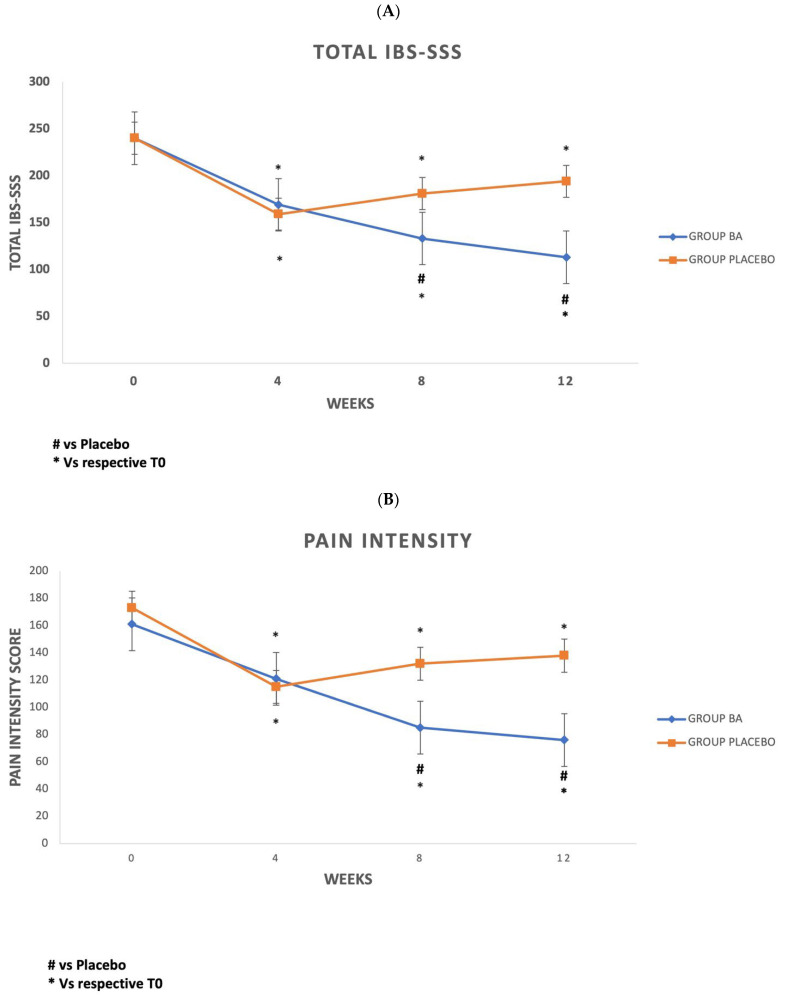

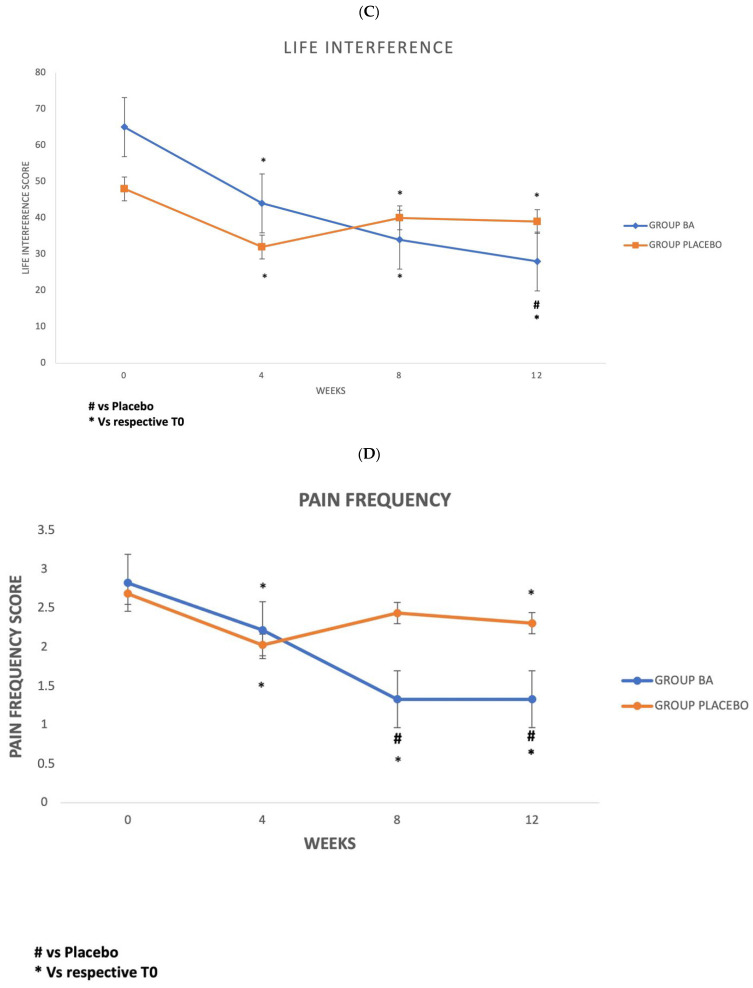
(**A**) IBS-Symptom Severity Scale (*IBS-SSS*) trend in *BA* and placebo group, (**B**) Pain Intensity Score (*PIS*) trend in *BA* and placebo group, (**C**) Life Interference Score (*LIS*) trend in *BA* and placebo group, (**D**) Pain Frequency Score *(PFS*) trend in *BA* and placebo group.

The *IBS-SSS*, *PIS*, *LIS*, and Pain Frequency Score trends are shown in Figure 4A–D.

**Figure 5:**
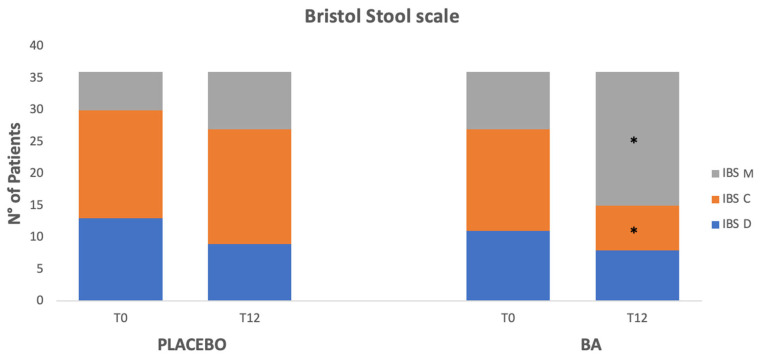
Bristol Stool Chart (*BSC*) subtypes in BA and in placebo groups at baseline and after 12 weeks. * Significant difference vs. T0.

Stool consistency was analyzed at T0 and the end of the treatment/placebo (T12) using the BSC. Among patients who received BA, at baseline BSC subtypes were 1–2 (constipation) in 44.5% (N 16), 3–4 (normal) in 25% (N 9), and 5–7 (diarrhea) in 30.5% (N 11). At the end of the treatment in the BA group, we found subtypes 1–2 in 19.4% (N 7), 3–4 in 58.3% (N 21), and 5–7 in 22.3% (N 8), with a significant decrease in BSC subtypes 1–2 (*p* = 0.02) and an increased percentage of BSC subtype 3–4 at the end of the treatment when compared with baseline (*p* = 0.004). In the placebo group, BSC subtypes were 1–2 in 47.2% (N17) at T0 vs. 50% (N18) at T12, subtypes 3–4 in 16.7% (N6) vs. 25% (N 9) at T12, and subtypes 5–7 in 36.1% (N13) at T0 vs. 25% (N9) at T12, with no significant changes in the BSC before and after 12 weeks of placebo *(*Figure 5).

### Safety

Both *BA* and placebo groups recorded no adverse events during treatment.

## 5. Discussion

This is the first study to demonstrate that *Bifidobacterium adolescentis PRL2019* may be effective in improving abdominal symptoms in children with *IBS* without adverse events.

In our study, 52.8% of *IBS* patients receiving *BA* achieved total remission of symptoms, compared with 19.4% in the placebo group (*p* < 0.05). We found that all considered *IBS* scores *(IBS-SSS*, *PIS*, *LIS*, *PFS*) significantly improved both in the *BA* and placebo group by the end of the treatment. Four weeks of treatment were sufficient to observe a significant reduction in all symptom scores in both groups. However, upon intergroup comparison, only the *BA* group showed a significant reduction in *IBS-SSS*, *PIS*, and *PFS* at T8 and T12 of *LIS* at T12 when compared with placebo outcomes. In contrast, no significant differences were observed between the two groups at T4 upon intergroup analysis. These data can be explained by considering the well-known placebo effect described in *IBS*.

Regarding bowel habit changes, the *BSC* improved in patients receiving *BA*, with a significantly higher percentage of *BSC* 3–4 subtypes at the end of treatment than at baseline. This was even more evident in patients showing *BSC* 1–2 (constipation) at T0 (Figure 5).

*IBS* is a chronic functional gastrointestinal disorder with a multifactorial and not yet well-known pathogenesis. *IBS* etiology correlates to gut dysbiosis, gastrointestinal motility disorder, intestinal infections, and visceral hypersensitivity [30]. Although the exact role of the intestinal flora is not entirely known, recent evidence has shown a possible regulating effect of some neurotransmitters, such us 5-hydroxytryptamine (*5-HT*), dopamine, g-aminobutyric acid (*GABA*), produced by some gut bacteria species, affecting the central nervous system (*CNS*) through the microbiota–gut–brain axis [31,32]. The two main species identified as *GABA* producers are Bifidobacterium and Lactobacillus [33], and, more specifically, only three *Bifidobacterium* species (*B. Dentium*, *B. Longum*, *B. adolescentis*) have been shown to produce *GABA* in in vitro studies [34]. In 2020, Duranti. et al. administered two *B. adolescentis* strains (PRL2019 and HD17T2H) in a mouse model, demonstrating a higher *GABA* expression in rats treated with these strains [23]. *GABA* is the primary mediator of inhibitory transmission in the mammalian central nervous system. It plays a pivotal role not only in psychological diseases, such as behavioral disorders, insomnia, and pain [35], but also in regulating some enteric nervous system (*ENS*) functions, such as acid secretion, gastric empties, intestinal motility, and pain perception [24]. Aggarwal et al. demonstrated that *GABA* levels are reduced in adult *IBS-D* patients compared with controls; furthermore, proinflammatory cytokines are upregulated in HT-29 cells (human colorectal adenocarcinoma cell line) treated with bicuculline methiodide (*GABA* antagonist) [25].

*GABA* receptors are distinguished into ionotropic (*GABA-A*, *GABA-C*) and metabotropic (*GABA-B*) receptors, abundantly expressed in the gastrointestinal tract, from the stomach to the ileum [36]. *GABA-B* receptors modulate vagal and spinal sensitivity at the spinal cord level and regulate several functions, such as gut motility and gut–brain signaling [24]. Although the exact role of GABAergic signaling on gut motility isn’t entirely known, it seems to influence the peristaltic reflex, both on ascending contractions and descending relaxation. More specifically, in 2014, Autieri et al. concluded that *GABA-A* agonists induced an excitatory effect on gut motility while *GABA-B* agonists had an inhibitory effect, suggesting an opposite contribution of these two types of receptors in modulating the ACh release, thus regulating the cholinergic component of the peristaltic reflex [37]. Furthermore, *GABA-A* receptors are usually activated in physiological conditions. At the same time, *GABA-B* seems to be activated in the presence of high concentrations of *GABA*, such as *GI* inflammations, which increase the release of enteric mediators [38]. This effect on the peristaltic reflex could explain the modification of bowel habits observed in our study in patients who received *BA*, especially in those showing an *IBS* constipation subtype at baseline (Figure 5).

Furthermore, there is experimental evidence about *GABA-B* receptors having an antinociceptive effect when *GABA-B* agonists, such as Baclofen, are co-administrated with morphine, and this is true also for drugs used to reduce visceral pain [39,40], suggesting that *GABA-B* receptors could have a role in regulating antinociceptive effects at the level of spinal cord, as well as in regulating both somatic and visceral nociceptive stimuli. Finally, *GABA* receptors seem involved in regulating several immunological processes, such as the downregulation of pro-inflammatory cytokines, since they are expressed in some immune cells, such as mast cells, T-cells, and dendritic cells [41]. These data could explain the improvement of severity and frequency of abdominal symptoms, evaluated through validated scores, observed in our study in *IBS* patients who received *BA (*Figure 4). However, direct evidence on this topic is lacking, necessitating caution.

In the last decades, the gastrointestinal microbiome has generated considerable interest due to its potential for shaping visceral sensation through direct effects, interactions with the intestinal immune system and host genetics or diet, and its role in regulating bidirectional communication between the gut and brain. As shown, GABAergic signaling has been identified through preclinical research as capable of altering not only neuroreceptor signaling but also 5-HT receptors and G-protein-coupled receptors, including protease-activated and cannabinoid receptors. Other mechanisms underlying visceral hypersensitivity include neurotransmitter/peptide-mediated hyperalgesia (e.g., serotonin, calcitonin gene-related peptide, substance P), guanylate cyclase C signaling, stress-induced activation or remodeling of the hypothalamic–pituitary–adrenal axis, alteration of descending pain pathways, and sensitization of spinal afferents. Exploring these interactions with a multiomics approach is a main aim for the near future, and starting from clinical observations such as the results of our study could support the importance of this research field [42].

Our study has several strengths. This is a multicentric, double-blind, placebo-controlled study. Until now, no studies have been published evaluating the efficacy of *Bifidobacterium adolescentis PRL2019* on *IBS* patients, neither in adults nor children. However, we also acknowledge some limitations. The sample size is small, and patients were all recruited from a tertiary care hospital, thus likely exhibiting a more severe *IBS* phenotype compared with the general population. Further research is needed to confirm our data on a larger sample. Moreover, beyond the baseline dietary advice provided to all patients, we did not use a standardized questionnaire to compare the diets of the two cohorts.

In conclusion, we demonstrated for the first time the efficacy and safety of *Bifidobacterium adolescentis PRL2019* in improving abdominal symptoms in children with *IBS*, positively affecting bowel habits in the constipated subtype. Given the challenges in managing *IBS*, our findings suggest that *BA* could be a practical therapeutic approach to better control the severity and frequency of gastrointestinal symptoms in children with *IBS*.

## Figures and Tables

**Table 1:** Baseline characteristics of *Bifidobacterium adolescentis PRL2019* (*BA*) and placebo groups.

		BA	Placebo
Total		36	36
Males N (%)		13 (36.1%)	17 (47.2%)
Age, years (mean ± SD)		11.9 ± 1.9	12.6 ± 1.8
IBS subtype	Diarrhea (%)	10 (27.8%)	12 (33.3%)
	Constipation (%)	10 (27.8%)	11 (30.6%)
	Mixed (%)	16 (44.4%)	13 (36.1%)
Severity	Mild (%)	12 (33.3%)	13 (36.1%)
	Moderate (%)	13 (36.1%)	13 (36.1%)
	Severe (%)	11 (30.6%)	10 (27.8%)
Bristol Stool Chart (BSC)	1–2 (%)	16 (44.4%)	17 (47.2%)
	3–4 (%)	9 (25.0%)	6 (16.7%)
	5–7 (%)	11 (30.6%)	13 (36.1%)

**Table 2:** IBS Symptom Severity Scale (*IBS-SSS*), Pain Intensity Score (*PIS*), Life Interference Score (*LIS*), and Pain Frequency Score (*PFS*) in *BA* and in placebo groups at T0, T4, T8, T12 (mean ± SD). * Significant differences between BA and placebo at the same T.

	BA	Placebo	*p*
IBS-SSS			
T0 (mean ± SD)	240.3 ± 119.9	239.6 ± 106.5	0.860
T4	168.8 ± 102.0	159.9 ± 91.0	0.806
T8	133.3 ± 129.3	180.6 ± 112.5	0.016 *
T12	113.2 ± 134.5	194.4 ± 116.4	0.001 *
Pain Intensity Score			
T0 (mean ± SD)	161.1 ± 79.2	172.9 ± 80.5	0.411
T4	120.8 ± 72.3	114.6 ± 73.3	0.613
T8	84.7 ± 96.4	131.9 ± 88.4	0.004 *
T12	76.4 ± 92.7	137.5 ± 81.4	0.001 *
Life Interference Score			
T0 (mean ± SD)	64.6 ± 41.1	47.9 ± 28.3	0.125
T4	43.7 ± 33.5	31.9 ± 21.2	0.285
T8	34.0 ± 35.9	39.6 ± 30.1	0.139
T12	27.8 ± 37.2	38.9 ± 28.3	0.015 *
Pain Frequency Score			
T0 (mean ± SD)	2.8 ± 1.0	2.7 ± 1.2	0.429
T4	2.2 ± 1.2	2.0 ± 1.0	0.382
T8	1.3 ± 1.3	2.4 ± 1.2	0.001 *
T12	1.3 ± 1.6	2.3 ± 1.3	0.005 *

## Data Availability

The original contributions presented in this study are included in the article. Further inquiries can be directed to the corresponding author.
